# Dietary Intake and Sources of Potassium and the Relationship to Dietary Sodium in a Sample of Australian Pre-School Children

**DOI:** 10.3390/nu8080496

**Published:** 2016-08-13

**Authors:** Siobhan A. O’Halloran, Carley A. Grimes, Kathleen E. Lacy, Karen J. Campbell, Caryl A. Nowson

**Affiliations:** Institute for Physical Activity and Nutrition Research (IPAN), School of Exercise and Nutrition Sciences, Deakin University, Geelong 3220, VIC, Australia; s.ohalloran@deakin.edu.au (S.A.O.); carley.grimes@deakin.edu.au (C.A.G.); katie.lacy@deakin.edu.au (K.E.L.); karen.campbell@deakin.edu.au (K.J.C.)

**Keywords:** dietary potassium, sodium:potassium ratio, children, salt, diet, food sources, Australia, dietary sodium

## Abstract

The aim of this study was to determine the intake and food sources of potassium and the molar sodium:potassium (Na:K) ratio in a sample of Australian pre-school children. Mothers provided dietary recalls of their 3.5 years old children (previous participants of Melbourne Infant Feeding Activity and Nutrition Trial). The average daily potassium intake, the contribution of food groups to daily potassium intake, the Na:K ratio, and daily serves of fruit, dairy, and vegetables, were assessed via three unscheduled 24 h dietary recalls. The sample included 251 Australian children (125 male), mean age 3.5 (0.19) (SD) years. Mean potassium intake was 1618 (267) mg/day, the Na:K ratio was 1.47 (0.5) and 54% of children did not meet the Australian recommended adequate intake (AI) of 2000 mg/day for potassium. Main food sources of potassium were milk (27%), fruit (19%), and vegetable (14%) products/dishes. Food groups with the highest Na:K ratio were processed meats (7.8), white bread/rolls (6.0), and savoury sauces and condiments (5.4). Children had a mean intake of 1.4 (0.75) serves of fruit, 1.4 (0.72) dairy, and 0.52 (0.32) serves of vegetables per day. The majority of children had potassium intakes below the recommended AI. The Na:K ratio exceeded the recommended level of 1 and the average intake of vegetables was 2 serves/day below the recommended 2.5 serves/day and only 20% of recommended intake. An increase in vegetable consumption in pre-school children is recommended to increase dietary potassium and has the potential to decrease the Na:K ratio which is likely to have long-term health benefits.

## 1. Introduction

Hypertension is one of the most preventable causes of stroke and cardiovascular disease (CVD) [1]. Concern exists regarding the prevalence of hypertension in children and adolescents and the prevailing tracking pattern of blood pressure (BP) across the life course [2]. In children, high dietary sodium intake is associated with increased BP [3,4] and lower sodium intake associated with reduced BP [5].

High dietary potassium is also associated with BP: in adults, potassium intake has a protective effect on BP, but evidence of the effect of potassium on BP in children is mixed. For example, in a Greek cross-sectional study of 606 7-to-15-year-olds, systolic blood pressure (SBP) was significantly positively associated with potassium intake [6]. Stronger longitudinal evidence comes from Geleijnse et al. in which six annual 24-h urine samples were collected from Dutch children and showed over a seven years period, mean SBP was lower when potassium intakes were higher [7]. Conversely, no association between children’s potassium intake and BP was found in a meta-analysis of three intervention trials and one cohort study [8].

Beyond the individual effects of either dietary sodium or potassium on BP, cross-sectional and longitudinal evidence in adults has shown that the molar sodium-to-potassium (Na:K) ratio is positively associated with BP [9,10] and is a predictor of cardiovascular risk [11]. For example, in 783 adults aged 50–75 years, a one unit decrease in the Na:K ratio was associated with a reduction in SBP of 1.8 mm Hg [10]. In addition, Cook et al. reported a 24% increase in CVD risk per Na:K unit in 2275 adults aged 30–54 years [11]. Few data describing the effects of the Na:K ratio on BP in children exist. One longitudinal Dutch study reported in 255 children aged 5–17 years, a greater yearly rise in SBP over seven years in those with a higher Na:K ratio [7]. A study from the United States conducted in 2185 girls aged 9–17 years, reported that, over a 10 years period, those with a K:Na ratio ≥0.8 had SBP levels lower than those with K:Na ratios below 0.6 [12]. The World Health Organisation (WHO) recommends a potassium intake which results in an optimal Na:K ratio of close to one [13].

In Australia, utilising one day of 24-h recalled dietary data, the 2011–2013 Australian Health Survey (AHS) reported an average dietary potassium intake of 2042 mg in children aged 2–3 years [14], which is close to the recommended adequate intake (AI). The adequate intake is used when a recommended dietary intake cannot be determined. The upper level of intake is the highest average daily nutrient intake level likely to pose no adverse health effects to almost all individuals in the general population 15 of 2000 mg/day. In contrast, the average intake of dietary sodium is reported as 1484 mg/day [14], which exceeds the recommended daily Upper Level of Intake (UL) of 1000 mg/day [15] for dietary sodium by ~50%. Accordingly, it is likely that Australian children’s Na:K ratios will be higher than the optimal ratio of 1. Regarding the Na:K ratio for food sources, only one cross-sectional study has examined the Na:K ratio of children’s primary food sources and showed in French children aged 2–14 years, processed foods such as, breads, cheeses, breakfast cereals and seasonings had higher average Na:K ratios, ranging from 5.6 to 16.4, compared to the Na:K ratios ranging from 0.1 to 0.3 for fruits, vegetables and dairy [16]. To our knowledge, this is the first Australian study to assess the molar Na:K ratio in children and of food sources.

The aims of the present study were to assess, utilising three days of 24-h dietary intake, in a sample of Australian pre-school children: (a) dietary intake and food sources of potassium; and (b) the molar Na:K ratio of both food consumed and key food groups.

## 2. Materials and Methods

The Melbourne Infant Feeding Activity and Nutrition Trial (InFANT) program, conducted during 2008–2010 within the major metropolitan city of Melbourne, Australia, was a cluster-randomised controlled trial involving first-time parents attending parents’ groups when their children were 3–20 months of age [17]. Individual parents were eligible to participate if they gave informed written consent, were able to communicate in English and were first-time parents. Each parent-child dyad represented one parent and their first-born. Anticipatory guidance on diet, infant feeding, and physical activity was delivered to the intervention group, whereas the control group received information only on child health and development. A detailed description of the program can be found elsewhere [17]. Eighty-six percent of eligible parents consented to participate (*n* = 542) [18]. Data from this study are drawn from the post-intervention follow-up when children were aged approximately 3.5 years. Data were excluded for participants lost to follow-up (*n* = 181) and those with no dietary recall (*n* = 100). Children with fewer than three complete dietary recalls at 3.5 years were excluded (*n* = 6). Outliers for total energy intakes were identified and excluded according to the criterion of mean ± 3 SDs (*n* = 4). This resulted in a sample size of 251 children. In addition, in a previous study utilising the same dataset, we assessed sodium intake and dietary sources of sodium. [19]. Findings from that study were used to assess the Na:K ratio for the sample and key food groups. The InFANT program was approved by the Deakin University Human Ethics Research Ethics Committee (ID number: EC 175-20078) and the Victorian Government Department of Human Services, Office for Children, Research Coordinating Committee.

### 2.1. 24-h Dietary Recall and Food Sources

Dietary intake was assessed by trained nutritionists by telephone-administered five pass 24-h recall with the child’s parent when children were approximately 3.5 years of age. All food and beverages consumed midnight to midnight on the day before the interview were reported [20]. To help parents estimate their child’s food consumption, study-specific food measurement books were provided [21] and, where possible, call days were unscheduled (96% of calls) [18]. Three days of dietary intake were assessed (recalls were non-consecutive and included one weekend day) and the mean number of days between the first and last recall was 15.5 days (SD 18.3 days) [18].

Potassium intake was calculated using the 2007 Australian nutrient composition database (AUSNUT 2007), where foods are classified using a hierarchical numeric system. Individual food and beverage items are assigned an eight-digit food ID where two-, three-, and five-digit food groups describe major, sub-major, and minor foods, respectively. The last three digits of the eight-digit food ID are sequentially assigned to foods once they have been grouped within the five-digit group [22]. A detailed list of the food group classification system can be found in AUSNUT 2007 http://www.foodstandards.gov.au/science/monitoringnutrients/ausnut/Pages/ausnut2007.aspx. [23].

### 2.2. Potassium and Sodium Intake Recommendations

Children’s potassium intake was compared to the Australian National Health and Medical Research Council (NHMRC) adequate intake (AI) for potassium of 2000 mg/day for children aged 1–3 years. The AI is the average daily nutrient level assumed to be adequate for a healthy population. An upper level (UL) of intake or a recommended dietary intake for potassium as not been set for children [15].

Children’s sodium intake was compared with the NHMRC UL of intake for sodium of 1000 mg/day (salt equivalent 2.5 g/day) for children aged 1–3 years and was presented in our previous paper [19]. The UL is defined as the highest average daily intake which is likely to pose no adverse effects [15].

The World Health Organisation (WHO) recommendation for a Na:K ratio of ≤1 was used when assessing the average molar Na:K [13].

### 2.3. Other Measures

Demographic and socioeconomic data was collected at baseline when children were three months of age, via self-administered paper-based questionnaires. Maternal education was dichotomised as low (secondary school or trade qualifications or less) or high (college or university or more) [21].

### 2.4. Data Analysis and Statistical Analyses

Descriptive statistics were used to describe food group contributions to total potassium and energy intakes and the Na:K ratio for the sample. Mean contributions and standard deviations (SD) for the sample, and the percentage of the sample consuming each food group were calculated.

To calculate the average daily contribution of each food group to the participants’ average daily potassium intake and participants’ average daily energy intake over three days, the mean ratio method at the individual level was used [24]. Sodium and potassium density were calculated as mg/1000 kJ to correct for differences in children’s energy intake.

The mean and SD for the molar sodium:potassium (Na:K) ratio were calculated using the average daily potassium intake and the average daily sodium intake from our previous study [19]. To calculate the average molar Na:K ratio, sodium and potassium in micrograms (mg) were converted to milli-moles (mmol) using the following conversion [15]:

23 mg sodium = 1 mmol sodium(1)

39 mg potassium = 1 mmol potassium (2)

#### 2.4.1. Na:K Ratio Key Food Groups

To calculate the mean Na:K ratio for food groups, the average amount of sodium and potassium across the three days of intake from each food group was calculated and converted to mmol using the above conversion.

#### 2.4.2. Serving Sizes

To assess the serves per day (as recommended by the Australian Dietary Guidelines [25]) of key food groups, the sample was divided into two groups: (i) mean potassium intake below the AI (*n* = 135); and (ii) mean potassium intake above AI (*n* = 116). The average intake across three days of intake for vegetable and fruit were calculated at the two-digit food group level. To account for the variation in the recommended serving sizes within the milk products/dishes food group, the average intake across three days of intake for these foods was calculated at the three-digit food group level (i.e., cheese, milk, yoghurt). To calculate the serves/day, the total intake in grams was divided by the recommended serving size (g) for each food group [25]. Differences in the serves/day of key food groups between the group below and above the AI for potassium were assessed using the standard error of the mean (±SEM) and the independent samples *t* test.

A *p* value of < 0.05 was considered significant. Analyses were conducted using StataSE 12 software (Release; StataCorpLP, College Station, TX, USA).

## 3. Results

### 3.1. Participants Lost to Follow-up

Demographically, those participants (mothers) lost to follow-up were less likely to be on maternity leave (e.g., when baseline data was collected), less educated, more likely married, born in Australia, and to speak English at home, and the children had a higher body mass index (BMI) *z*-score, compared to those retained. One father (within the lost to follow-up group) with incomplete dietary data was excluded from this study.

### 3.2. InFANT Follow-up Participants

Demographic characteristics of the sample are shown in Table 1. Two-hundred fifty-one children, who were approximately 3.5 years of age with an equal gender distribution, were included. The intervention and the control groups were combined for analysis as there was no difference in dietary potassium intake between the groups; 2024 (SD 514) mg/day potassium (52 (13.1) mmol/day), and 1943 (483) mg/day potassium (50 (12.3) mmol/day), respectively (*p* = 0.20).

The average molar Na:K ratio for the group was 1.3 (0.45), with 77% of children above the WHO-recommended Na:K of one [14] (Table 1). The average molar Na:K ratio for boys and girls were similar; 1.32 and 1.35, respectively (*p* = 0.6). The average daily potassium intake in boys 2061 (±SEM 45) mg/day (53 (1) mmol) was ~8% higher than girls; 1907 (43) mg/day (49 (1) mmol), (*p* = 0.01). However, there was no difference between the average daily sodium intake for boys; 1565 (50) mg/day (68 (2) mmol/day) and girls; 1452 (32) mg/day (63 (2) mmol/day) (*p* = 0. 07). Fifty-four percent (*n* = 135) of children did not achieve the recommended AI for potassium of 2000 mg/day for children aged 1–3 years (Table 2). The Na:K ratio for the group above the AI was 1.18, compared to 1.47 for the group below the AI (*p* ≤ 0.001). Compared with children of high social economic position (SEP) (as defined by parental education status), children of low SEP had a significantly higher Na:K ratio but not a significantly higher average daily sodium or potassium intake (data not shown).

### 3.3. Food Group Contributions to Potassium

Dairy milk and yoghurt provided approximately one quarter of total potassium, and approximately 17% of energy consumed (Table 3). Tropical fruits and potatoes were also important sources of potassium and contributed 7% and 6%, respectively, but less than 5% of total energy intake. Within the cereal/cereal products category, breads and bread rolls, and breakfast cereal and bars collectively contributed ~8% of potassium and ~13% of energy.

### 3.4. Food Group Na:K Ratio

Food groups providing the highest Na:K ratio were processed meats, white bread/rolls, and savoury sauces and condiments, with Na:K ratios of 7.8, 6.0, and 5.4, respectively (Table 3). Other food groups with a high Na:K ratio included mixed grain bread/rolls (Na:K ratio 4.2), mixed dishes where cereal is the major ingredient (Na:K ratio 3.5) and soup (Na:K ratio 2.8). Lowest food group Na:K ratios were observed for fruit products/dishes, vegetable products/dishes, and milk product dishes, all of which had a Na:K ratio of less than 1.

### 3.5. Serving Sizes of Food Groups

Over the course of the three days of food intake, children above the AI for potassium met the recommended serves/day for dairy and fruit. Those children with a mean potassium intake below the recommended AI intake met the recommended serves/day for fruit only. In addition, those children in the group with an average potassium intake above the AI consumed significantly more fruits, vegetables, and dairy compared to the group with an average potassium intake below the AI (Table 4).

## 4. Discussion

In this sample, across three days of intake, the average dietary potassium intake in Australia pre-school children was 1983 mg/day, which was close to the National Health Medical Research Council (NHMRC) adequate intake (AI) of 2000 mg/day for potassium for children aged 1–3 years [15]. Our findings are consistent with the most recent national 2011–2013 Australian Health Survey (AHS) which reported, across one day of intake, an average daily potassium intake of 2042 mg/day in children aged 2–3 years [14]. Within our group, key contributors to potassium were dairy (specifically milk and yogurt), fruit (specifically tropical fruit), and vegetables (specifically potatoes). In general, our results corresponded well with the 2011–2013 AHS survey data for children aged 2–3 years which also reported milk, tropical fruit, and potatoes as key contributors to potassium, providing 21%, 6%, and 5%, of potassium intakes, respectively [14].

Importantly, more than half of the children within our sample did not achieve the recommended AI of 2000 mg/day for potassium [15]. These low potassium intakes appear to be driven by children’s low vegetable consumption given these children’s intakes fell well below the Australian daily targets for this age group (average 0.5 serves versus recommended 2.5 serves [25]). These low vegetable intakes were consistent (though consumption was slightly higher) even in the group achieving potassium AIs. Higher potassium intakes resulted primarily by additional fruit rather than vegetable consumption. Generally, our findings corresponded well with national data, confirming low vegetable consumption amongst Australian pre-school children; the 2011–2013 AHS reported an average of ~1.3 serves vegetables, compared to recommended 2.5 serves/day. In contrast, the AHS reported an average of 1.9 serves of dairy, and 1.8 serves of fruit; thus, children met the recommended serves of 1.5 of dairy and 1 of fruit, respectively [27]. Dairy is an important source of potassium and as young children in our sample did not quite meet the recommended 1.5 serves/day, it is important that dairy foods are encouraged in young children’s diets.

In our group, milk products/dishes were key sources of both sodium and potassium. Other main sources of sodium were cereal/products and meat poultry whereas major sources of potassium were fruit and vegetables. Therefore, it is possible for Australian pre-school children to attain the optimal Na:K ratio of one [13] by concomitantly increasing their intake of potassium-rich foods and decreasing foods high in sodium. To achieve this, we suggest two key approaches; the first approach involves promoting food choice to increase pre-school children’s daily vegetable consumption (consistent with current Australian dietary guidelines [25]). Early childhood is an important time to establish healthy dietary behaviours, such as vegetable consumption, as dietary habits established in young children follow a trajectory into adulthood [28]. Furthermore, large prospective studies have shown higher intakes of vegetables during childhood were associated with a lower risks of stroke [29] and cancer risk [30] in adulthood. Increasing vegetable consumption may also be important for preventing childhood obesity [31]. Thus, an increased vegetable consumption may not only reduce the Na:K ratio (improving risk for high BP), but will also likely result in a decrease of other CVD risk, as well as a range of other health benefits. Currently, there are no interventions which specifically target increasing vegetable intake to alter the Na:K ratio in children. Given Australian children’s vegetable intakes are low, targeting a wide-range of settings (e.g., home, childcare centres) [32] are needed to alter children’s Na:K ratio closer to the recommendations.

The second approach to lower Australian pre-school children’s Na:K ratio is to reduce children’s sodium intakes. Given sodium in young children’s diets is provided mostly by breads and cereals, processed meats, and cheese [14], meeting sodium recommendations at the individual level is difficult to achieve and reformulation of these products with lower sodium content is required. In Australia, despite voluntary sodium reduction targets across a number of food categories [33], the average Australian adult’s salt intake remains excessive (an average salt intake 7.8 g/day) [34]. In contrast, in the United Kingdom the salt content across a number of food categories has been reduced by up to 57%, resulting in a 1.4 g/day fall in mean population salt intake [35]. Thus, more effective salt reduction targets from the Australian food industry to reformulate high sodium food products is required.

Within our sample, Na:K ratios exceeded the WHO recommendation of 1 [13], even in the group of children who exceeded the AI potassium recommendation. Few data are available reporting the Na:K ratio in children, with studies limited to North America and Europe. For example, an average Na:K ratio of 1.03 (SE 0.01) was reported for American children aged 1–3 years [36]. Amongst older Spanish children (6–14 years) and 2–14 years old French children, the average reported Na:K ratio was 3.6 (SD ± 1.3) [37] and 1.64 (range: 1.61–1.67) [16], respectively. Importantly, in our study children from both groups (above and below the AI), also consumed sodium amounts which exceeded the recommended upper level (UL) of 1000 mg/day [15] which, consequently, appeared to drive up the Na:K ratio. If children were to lower their sodium intake, to the UL amount, their Na:K ratios would fall closer to a ratio of 1, even in those with a potassium intake below recommendations.

In addition to assessing the Na:K ratio for our sample, we also identified the main food groups with high Na:K ratios (e.g., processed meats, savoury sauces/gravies, bread/bread rolls). These processed foods, as outlined earlier, are key dietary sources of sodium in young children and as an estimated three quarters of dietary sodium in developed countries comes from processed foods [38], our findings reaffirm the need for sodium content reformulation by the food industry.

It is important to consider the strengths and limitations of this study. The three day, multi-pass 24-h recall allowed for a robust assessment of usual potassium intake and the disaggregation of the recipes allowed the food sources of potassium to be fully described. However, the results of this study cannot be generalised to the Australian population in this age group as the parents who participated in this study were more likely to speak English at home, be Australian born, have a university qualification, and their children had a lower body mass index (BMI) *z*-score. Children from a lower socio-economic position (SEP) tend to have lower intakes of fruits and vegetables [39]. Thus, our findings likely represent the best case scenario given the SEP variation in healthy diets. Another limitation of our study concerns the estimation of serves/day of vegetables, dairy, and fruit. Some mixed dishes (i.e., a vegetable curry) were coded using AUSNUT 2007 [23] rather than using a recipe approach, which may impact the estimation of dairy/vegetable/fruit consumption. In addition, the Na:K ratio in our sample is likely to be higher as the 24-h recall method does not capture the use of salt added at the table or in cooking and, thus, is likely to under-represent the true value of salt intake.

## 5. Conclusions

Our study describes total intakes and key food sources of dietary potassium and the molar Na:K ratio of both food consumed and food groups in a sample of Australian pre-school children. We have demonstrated that Australian pre-school children, on average, have a high Na:K ratio driven by the excessive amounts of sodium contained in manufactured foods and low dietary potassium intakes due to vegetable consumption below recommendations. A more widespread reformulation of high sodium foods and an increase in the number of vegetable serves/day is required to improve young children’s Na:K ratios.

## Figures and Tables

**Table 1:** Characteristics for children and mothers who participated in follow-up data collection when the children were 3.5 years old.

**Child Characteristics**		
Sex	*n*	%
Boys	125	50
Girls	126	50
Demographics	Mean	SD
Age (years)	3.6	0.41
Weight (kg)	16.6	2.0
Height (cm)	100.7	4.0
Body Mass Index *z*-score ^1^	0.6	0.8
**Mothers’ Characteristics**	*n*	%
Employment status ^2^		
On maternity leave	178	71
Employed full time	3	1
Employed part time	19	8
Unemployed	8	3
Student	2	1
Home duties	36	14
Other	5	2
Highest level of education		
Bachelor degree or higher	158	63
Trade or high school	93	37
Marital status		
Partner	249	99
Separated	1	0.5
Single parent	1	0.5
Country of birth		
Australia	208	83
Other	43	17
Main language at home		
English	240	96
Other	11	4
**Sodium (Na) and Potassium (K)**	Mean	SD
Daily K intake (mg/day)	1983	499
Daily K intake (mmol/day)	51	21
Daily K density (mg/1000 kJ)	383	65
Daily Na intake (mg/day) ^3^	1508	495
Daily Na intake (mmol/day) ^3^	65	13
Daily Na density (mg/day) ^3^	290	70
Daily molar Na:K ratio	1.3	0.45

^1^ Body mass index *z*-scores were calculated by using World Health Organization gender-specific BMI-for-age growth charts [26]; ^2^ Data collected at baseline when children were three months old; ^3^ Average daily sodium intakes from our previous study [19].

**Table 2:** Descriptive characteristics for sodium (Na), potassium (K), and energy intakes for the two groups of children above and below the recommended adequate intake (AI) of 2000 mg/day for potassium for children aged 1–3 years [15] (mean (SD)).

	Above AI (*n* = 116)	Below AI (*n* = 135)	*p* Value ^1^
Daily K intake (mg/day)	2409 (345)	1618 (267)	<0.001
Daily K intake (mmol/day)	62 (9)	41 (7)	<0.001
Daily Na intake (mg/day)	1663 (507)	1379 (450)	<0.001
Daily salt equivalent (g/day)	4.15 (1.2)	3.4 (1.1)	<0.001
Daily Na intake (mmol/day)	72 (22)	60 (19)	<0.001
Na:K ratio	1.18 (0.3)	1.47 (0.5)	<0.001
Energy intake (kJ/day)	5912 (870)	4571 (775)	<0.001

^1^ Means are compared between above and below AI using independent *t* test.

**Table 3:** The main food sources of sodium (Na), potassium (K), energy, and Na:K ratio from major, sub-major, and minor food categories in Australian pre-school children (food group categories that contribute >1.0% of potassium to daily intake). The Na:K ratio of major food categories is shown in descending order.

Food Group Name [15]	Proportion Consuming (%)	Contribution to Energy Intake (%)	Contribution to Na Intake (%)	Contribution to K Intake (%)	Na:K Ratio (mmol)
**Savoury sauces and condiments ^1^**	63	0.7	3.8	1	5.4
Gravies And Savoury Sauces ^2^	53	0.5	3.1	1	4.4
**Cereal based products/dishes**	99	13	15	4.8	4.3
Mixed Dishes Where Cereal Is The Major Ingredient	43	3.5	5.4	2.1	3.5
*Savoury Pasta/Noodle And Sauce Dishes ^3^*	*19*	*1.3*	*2*	*1*	*2.7*
**Cereals and cereal products**	100	23.1	25.4	11.3	2.8
Regular bread and bread rolls	97	10.3	17	5	4.3
*Breads, And Bread Rolls, White*	*50*	*2.8*	*4.4*	*1*	*6*
*Breads, And Bread Rolls, Mixed Grain*	*51*	*2*	*3.5*	*1*	*4.2*
*Breads, And Bread Rolls, Wholemeal*	*31*	*2.9*	*4.8*	*1.8*	*3.4*
Breakfast cereals and bars	78	4.9	4	2.9	1.7
*Breakfast Cereal, Wheat Based Fortified*	*50*	*2.8*	*2.1*	*1.8*	*1.4*
Breakfast cereal hot porridge	19	0.5	0.4	1.3	0.3
*Breakfast Cereal, Hot Porridge Type, Made From Oats (Including plain, flavoured*	*19*	*0.5*	*0.41*	*1.3*	*0.3*
**Meat, poultry and game products and dishes**	96	9.3	17.5	8.6	2.8
*Beef*	*24*	*0.2*	*0.3*	*1*	*0.5*
*Muscle meat*	*57*	*0.6*	*0.6*	*1.7*	*0.4*
Processed meat	56	0.5	8.1	1.5	7.8
*Ham*	*39*	*0.5*	*5*	*1*	*7.8*
Poultry and Feathered Game	39	0.3	0.4	1.1	0.4
*Chicken*	*39*	*0.3*	*0.4*	*1.1*	*0.4*
Mixed Dishes Where Poultry or Game Is The Major Component	35	1.4	2.2	1.1	2.7
Mixed Dishes Where Beef, Veal or Lamb Is The Major Component	37	1.6	1.8	2.1	1
*Beef, or Veal Stew, Casserole, Stir Fry With Gravy or Sauce Only*	*31*	*0.4*	*1.2*	*1.7*	*0.8*
**Soup**	17	0.6	2	1	2.8
Soup (Prepared, Ready to Eat)	16	0.6	2	1	2.8
**Miscellaneous**	52	0.2	3.3	1.7	2.6
Yeast, Yeast, Vegetable And Meat Extracts	48	0.2	2.8	1.7	2.2
*Yeast, Vegetable And Meat Extracts*	*48*	*0*	*2.8*	*1.7*	*2.2*
**Fish and Seafood Products**	39	1.6	2.1	1.6	1.6
**Milk products and dishes**	100	24	19.3	27.4	0.9
Dairy milk	94	13.1	7.2	19.1	0.4
*Milk, Cow, Fluid, Regular Whole, Full Fat*	*81*	*3.6*	*5.5*	*14.7*	*0.4*
*Milk, Cow, Fluid, Reduced Fat,* <*2%*	*23*	*0.6*	*1.3*	*3.3*	*0.4*
Yoghurt	76	4	2.2	5.8	0.4
*Yoghurt, Flavoured or Added Fruit, Full Fat*	*37*	*0.6*	*0.9*	*2.5*	*0*
*Yoghurt, Full Fat, Fruit and/or Cereal Added*	*14*	*0.2*	*0.27*	*1*	*0.3*
*Yoghurt, Flavoured or Added Fruit, Reduced Fat*	*23*	*0.3*	*0.54*	*1.3*	*0.5*
**Dairy Substitutes**	10	0.2	0.9	1.3	0.8
Dairy Milk Substitutes, Unflavoured	9	0.2	0.9	1.2	0.4
*Soy-Based Beverage, Plain, Fortified*	*7*	*0.1*	*0.5*	*1*	*0.6*
**Confectionary and Cereal/Nut/Fruit/Seed bars**	69	0.8	0.6	1	0.6
**Vegetable products/dishes**	98	5.4	3.3	13.8	0.3
Potatoes	67	3	1.4	5.7	0.1
*Potatoes*	*35*	*0.2*	*0*	*2.4*	*0*
*Potato Products (*e.g., *potato chips/wedges, gems and hash browns)*	*29*	*0.6*	*1.2*	*2.4*	*0.6*
Other Vegetables And Vegetable Combinations	55	0.2	0.4	1	0.5
Carrot And Similar Root Vegetables	68	0	0.2	1.2	0.2
*Carrot And Similar Root Vegetables*	*68*	*0*	*0.2*	*1.2*	*0.2*
Cabbage, Cauliflower And Similar Brassica Vegetables	45	0.5	0.2	1.1	0.1
*Cabbage, Cauliflower And Similar Brassica Vegetables*	*45*	*0*	*0.2*	*1.1*	*0.1*
**Non-alcoholic beverages**	66	0.6	0.5	2.8	0.2
Fruit and Vegetable Juices and Drinks	40	0.4	0.2	2.5	0.1
*Fruit Juices*	37	0.3	0.1	2.1	0
**Fruit Products/Dishes**	99	3.1	0.4	19.4	0
Berry	54	0.1	0.7	1.2	0
*Berry Fruit*	*54*	*0.1*	*0*	*1.1*	*0*
Stone Fruit	19	0.1	0	1.4	0
*Other Stone Fruit*	*6*	*0.1*	*0*	*1*	*0*
Tropical Fruit	71	1	0	7	0
*Bananas*	*67*	*0.8*	*0*	*6.4*	*0*
Other Fruit	65	0.5	0.1	3.8	0
Dried fruit, preserved fruit	35	0.3	0	1.9	0
*Dried Vine Fruit*	*29*	*0.2*	*0*	*1.3*	*0*
Other fruiting vegetables	66	0.3	0.1	2.3	0
*Other Fruiting Vegetables*	*55*	*0.3*	*0.1*	*1.8*	*0*
Other ^4^	-	17.4	5.9	4.3	-

^1^ Bold text denotes major food category; ^2^ Normal text denotes sub-major food category; ^3^ Italic text denotes minor food category; ^4^ Other includes food with less than 1% potassium.

**Table 4:** Differences in serves of food groups by participants who did not meet the recommended AI for potassium (mean (± SEM)).

	Below AI	±SEM	Above AI	±SEM	*p* = Value ^1^	Recommended Serves/Day
	*n* = 135 (54%)		*n* = 116 (46%)			
Potassium Intake (mg/day)	1618	23	2409	32	<0.001	
Food group						
Fruit serves	1		1.6		0.001	1
Vegetables serves	0.4		0.6		<0.001	2.5
Dairy serves	1.2		1.6		<0.001	1.5

Recommended adequate intake (AI) for potassium of 2000 mg/day [15]. ^1^ Difference between those below and above the AI using the independent *t* test. Recommended serving weights: 1 serve fruit = 150 g; 1 serve vegetable = 75 g; 1 serve of dairy = 250 mL milk; 120 mL evaporated milk; 40 g hard cheese; 120 g ricotta cheese; 200 g yoghurt; and 250 mL soy/rice milk [26].
